# Poly[diaqua-μ_2_-oxalato-di-μ_4_-terephthalato-diytterbium(III)]

**DOI:** 10.1107/S1600536810033052

**Published:** 2010-08-25

**Authors:** Chun-Xiang Wang, Zhi-Feng Li

**Affiliations:** a School of Materials & Chemical Engineering, Jiangxi University of Science and Technology, Ganzhou 341000, People’s Republic of China

## Abstract

The crystal structure of the title complex, [Yb_2_(C_8_H_4_O_4_)_2_(C_2_O_4_)(H_2_O)_2_]_*n*_, features an extended three-dimensional framework made up of Yb^3+^ ions coordinated by terephthalate ligands, oxalate ligands and water mol­ecules. The Yb^3+^ ion has a distorted square-anti­prismatic coordination formed by one aqua ligand, two O atoms from an oxalate ligand and five O atoms belonging to four terephthalate anions. Two symmetry-independent terephthalate anions, as well as the oxalate anion, occupy special positions on inversion centers. The water molecule participates in O—H⋯O hydrogen bonding with both terephthalate anions.

## Related literature

For isotypic structures, derivatives of Lu and Dy, see: Li & Wang (2009 ▶) and Li *et al.* (2009 ▶), respectively.
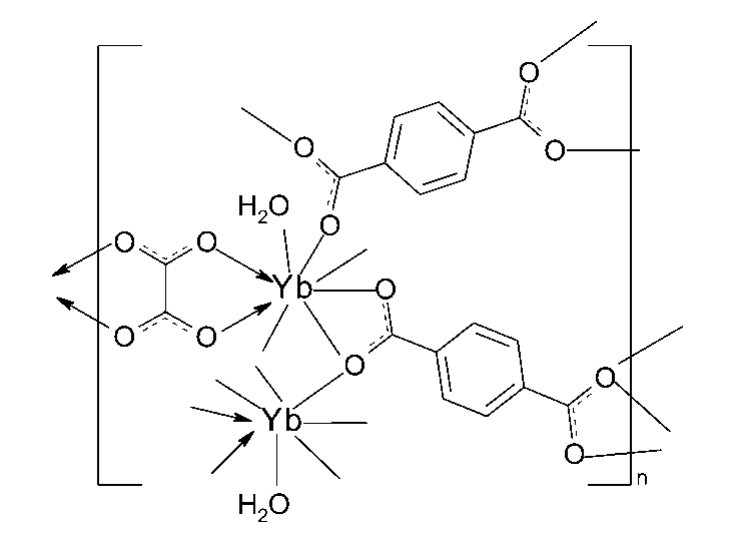


## Experimental

### 

#### Crystal data

[Yb_2_(C_8_H_4_O_4_)_2_(C_2_O_4_)(H_2_O)_2_]
*M* *_r_* = 798.36
Triclinic, 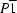

*a* = 7.034 (2) Å
*b* = 7.583 (2) Å
*c* = 10.213 (3) Å
α = 75.372 (4)°
β = 70.851 (4)°
γ = 88.126 (4)°
*V* = 497.2 (2) Å^3^
*Z* = 1
Mo *K*α radiation
μ = 9.43 mm^−1^
*T* = 295 K
0.24 × 0.15 × 0.05 mm

#### Data collection

Bruker SMART APEXII CCD area-detector diffractometer
Absorption correction: multi-scan (*SADABS*; Sheldrick, 2003 ▶) *T*_min_ = 0.211, *T*_max_ = 0.629
2630 measured reflections
1882 independent reflections
1785 reflections with *I* > 2σ(*I*)
*R*_int_ = 0.025

#### Refinement

*R*[*F* ^2^ > 2σ(*F* ^2^)] = 0.046
*wR*(*F* ^2^) = 0.120
*S* = 1.06
1882 reflections
154 parameters
H-atom parameters constrained
Δρ_max_ = 4.45 e Å^−3^
Δρ_min_ = −4.21 e Å^−3^

### 

Data collection: *APEX2* (Bruker, 2004 ▶); cell refinement: *SAINT* (Bruker, 2004 ▶); data reduction: *SAINT*; program(s) used to solve structure: *SHELXS97* (Sheldrick, 2008 ▶); program(s) used to refine structure: *SHELXL97* (Sheldrick, 2008 ▶); molecular graphics: *SHELXTL* (Sheldrick, 2008 ▶); software used to prepare material for publication: *SHELXTL*.

## Supplementary Material

Crystal structure: contains datablocks I, global. DOI: 10.1107/S1600536810033052/ya2126sup1.cif

Structure factors: contains datablocks I. DOI: 10.1107/S1600536810033052/ya2126Isup2.hkl

Additional supplementary materials:  crystallographic information; 3D view; checkCIF report

## Figures and Tables

**Table 1:** Selected bond lengths (Å)

Yb—O1	2.790 (6)
Yb—O1^i^	2.315 (6)
Yb—O2	2.314 (6)
Yb—O3	2.264 (6)
Yb—O4^ii^	2.209 (6)
Yb—O5	2.310 (6)
Yb—O6^iii^	2.321 (6)
Yb—O7	2.293 (5)

Symmetry codes: (i) 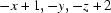; (ii) 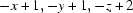; (iii) 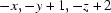.

**Table 2:** Hydrogen-bond geometry (Å, °)

*D*—H⋯*A*	*D*—H	H⋯*A*	*D*⋯*A*	*D*—H⋯*A*
O7—H7*A*⋯O3^i^	0.85	1.92	2.751 (6)	167
O7—H7*B*⋯O2^iv^	0.85	1.91	2.754 (6)	178

Symmetry codes: (i) 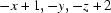; (iv) 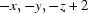.

**Fig. 1.**
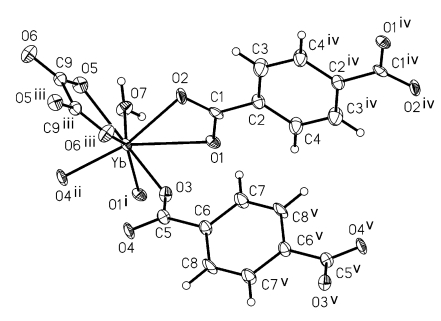
The fragment of the structure of the title compound, with the atom-numbering scheme. Displacement ellipsoids are drawn at the 30% probability level and H atoms are shown as small circles of arbitrary radii. Symmetry codes: (i) 1 -x, -y, 2 - z; (ii) 1 - x, 1 - y, 2 - z; (iii) -x, 1 - y, 2 - z; (iv) 1 - x, -y, 1 - z; (v) 2 - x, 1 - y, 1 - z.

## supplementary crystallographic information

### Comment

The title compound is isostructural with its Lu and Dy analogues (Li & Wang, 2009; Li *et al.*, 2009). As illustrated in Fig. 1, the Yb atoms is coordinated by two oxalate O atoms and five O atoms of four terephthalate anions; an aqua ligand completes a distorted square antiprismatic geometry. The Yb-O distances are in the range of 2.208 (6)–2.790 (6) Å, with an average Yb-O bond of 2.352 Å; these parameters are similar to M-O bonds found in the above mentioned previously reported isostructural complexes.

In the title complex, both symmetry independent terephthalate (tp) anions occupy special positions on the inversion centers; nevertheless they exhibit different modes of coordination of Yb atoms. The tp1 anion (O1 to O2, C1 to C4) functions as chelating-bridging tridentate ligand: two carboxylate oxygen atoms (O1 and O2) chelate one Yb atom; the O1 atom is additionally bonded to another Yb atom; the Yb···Yb separation is 4.232 (1) Å. Two edge-sharing [YbO_8_] polyhedra are bridged by the bidentate tp2 (O3 to O4, C5 to C8) ligands thus generating chains along the [010] direction. The chains are further linked by the tp1 and tp2 ligands into three-dimensional framework. The oxalate anion is also located on the inversion center and acts as a tetradentate ligand connecting the edge-sharing [YbO_8_] polyhedra along the [100] direction thus even further stabilizing the three-dimensional framework. The aqua ligand provides H-bond donors which participate in H-bonds with terephthalate oxygen atoms O2 and O3.

### Experimental

A mixture of YbCl_3_.6H_2_O (1.00 mmol, 0.39 g), oxalic acid (0.50 mmol, 0.05 g), terephthalic acid (0.50 mmol, 0.09 g), NaOH (2.00 mmol, 0.08 g) and H_2_O (10.0 ml) was heated in a 23 ml stainless steel reactor with a Teflon liner at 453 K for 72 h. A small amount of colorless plate-like crystals were filtered and washed with water and acetone.

### Refinement

H atoms attached to C atoms were included at calculated positions and treated as riding atoms [C–H = 0.93 Å and *U*_iso_(H) = 1.2*U*_eq_(C)]. Water H atoms were located in difference Fourier maps placed at the idealized positions with O-H distance of 0.85 Å and included in the final refinement as fixed contribution with *U*_iso_(H) = 1.5*U*_eq_(O). The highest density peak and deepest hole are located at 0.87 Å and 1.09 Å from the Yb atom respectively.

### Crystal data

**Table:**

[Yb_2_(C_8_H_4_O_4_)_2_(C_2_O_4_)(H_2_O)_2_]	*Z* = 1
*M**_r_* = 798.36	*F*(000) = 372
Triclinic, *P*1	*D*_x_ = 2.666 Mg m^−^^3^
Hall symbol: -p 1	Mo *K*α radiation, λ = 0.71073 Å
*a* = 7.034 (2) Å	Cell parameters from 196 reflections
*b* = 7.583 (2) Å	θ = 2.1–27.3°
*c* = 10.213 (3) Å	µ = 9.43 mm^−^^1^
α = 75.372 (4)°	*T* = 295 K
β = 70.851 (4)°	Plate, colorless
γ = 88.126 (4)°	0.24 × 0.15 × 0.05 mm
*V* = 497.2 (2) Å^3^	

### Data collection

**Table:**

Bruker SMART APEXII CCD area-detector diffractometer	1882 independent reflections
Radiation source: fine-focus sealed tube	1785 reflections with *I* > 2σ(*I*)
graphite	*R*_int_ = 0.025
φ and ω scans	θ_max_ = 26.0°, θ_min_ = 2.2°
Absorption correction: multi-scan (*SADABS*; Sheldrick, 2003)	*h* = −8→8
*T*_min_ = 0.211, *T*_max_ = 0.629	*k* = −8→9
2630 measured reflections	*l* = −7→12

### Refinement

**Table:**

Refinement on *F*^2^	Primary atom site location: structure-invariant direct methods
Least-squares matrix: full	Secondary atom site location: difference Fourier map
*R*[*F*^2^ > 2σ(*F*^2^)] = 0.046	Hydrogen site location: inferred from neighbouring sites
*wR*(*F*^2^) = 0.120	H-atom parameters constrained
*S* = 1.06	*w* = 1/[σ^2^(*F*_o_^2^) + (0.0995*P*)^2^] where *P* = (*F*_o_^2^ + 2*F*_c_^2^)/3
1882 reflections	(Δ/σ)_max_ < 0.001
154 parameters	Δρ_max_ = 4.45 e Å^−^^3^
0 restraints	Δρ_min_ = −4.21 e Å^−^^3^

### Special details

**Table:**

Geometry. All e.s.d.'s (except the e.s.d. in the dihedral angle between two l.s. planes) are estimated using the full covariance matrix. The cell e.s.d.'s are taken into account individually in the estimation of e.s.d.'s in distances, angles and torsion angles; correlations between e.s.d.'s in cell parameters are only used when they are defined by crystal symmetry. An approximate (isotropic) treatment of cell e.s.d.'s is used for estimating e.s.d.'s involving l.s. planes.
Refinement. Refinement of *F*^2^ against ALL reflections. The weighted *R*-factor *wR* and goodness of fit *S* are based on *F*^2^, conventional *R*-factors *R* are based on *F*, with *F* set to zero for negative *F*^2^. The threshold expression of *F*^2^ > σ(*F*^2^) is used only for calculating *R*-factors(gt) *etc*. and is not relevant to the choice of reflections for refinement. *R*-factors based on *F*^2^ are statistically about twice as large as those based on *F*, and *R*- factors based on ALL data will be even larger.

### Fractional atomic coordinates and isotropic or equivalent isotropic displacement parameters (Å^2^)

**Table:**

	*x*	*y*	*z*	*U*_iso_*/*U*_eq_	
Yb	0.31071 (4)	0.22186 (3)	1.01498 (3)	0.01504 (19)	
C1	0.3854 (12)	0.0294 (11)	0.7912 (9)	0.0203 (16)	
C2	0.4409 (12)	0.0093 (11)	0.6438 (9)	0.0202 (16)	
C3	0.3229 (16)	0.0805 (15)	0.5592 (11)	0.033 (2)	
H3	0.2048	0.1355	0.5976	0.039*	
C4	0.6198 (14)	−0.0700 (13)	0.5823 (10)	0.030 (2)	
H4	0.7015	−0.1163	0.6370	0.036*	
C5	0.7144 (12)	0.4806 (10)	0.7821 (9)	0.0189 (16)	
C6	0.8607 (13)	0.4930 (11)	0.6352 (10)	0.0211 (18)	
C7	0.8292 (13)	0.3852 (12)	0.5521 (10)	0.0282 (19)	
H7	0.7149	0.3066	0.5875	0.034*	
C8	1.0353 (13)	0.6070 (12)	0.5820 (10)	0.0275 (19)	
H8	1.0602	0.6780	0.6374	0.033*	
C9	−0.0833 (11)	0.4309 (10)	1.0574 (9)	0.0194 (16)	
O1	0.4818 (8)	−0.0459 (8)	0.8752 (6)	0.0228 (12)	
O2	0.2462 (9)	0.1353 (8)	0.8314 (6)	0.0228 (12)	
O3	0.6140 (8)	0.3311 (8)	0.8501 (6)	0.0224 (12)	
O4	0.6994 (9)	0.6187 (8)	0.8296 (7)	0.0248 (13)	
O5	−0.0295 (9)	0.2697 (8)	1.0944 (7)	0.0246 (12)	
O6	−0.2511 (8)	0.4911 (8)	1.1074 (7)	0.0254 (13)	
O7	0.1351 (8)	−0.0452 (7)	1.1590 (7)	0.0242 (13)	
H7B	0.0166	−0.0740	1.1644	0.036*	
H7A	0.1979	−0.1429	1.1672	0.036*	

### Atomic displacement parameters (Å^2^)

**Table:**

	*U*^11^	*U*^22^	*U*^33^	*U*^12^	*U*^13^	*U*^23^
Yb	0.0191 (3)	0.0156 (3)	0.0129 (3)	0.00119 (15)	−0.00688 (17)	−0.00586 (17)
C1	0.024 (4)	0.017 (4)	0.023 (5)	−0.008 (3)	−0.009 (3)	−0.007 (3)
C2	0.026 (4)	0.020 (4)	0.016 (4)	0.003 (3)	−0.008 (3)	−0.004 (3)
C3	0.036 (5)	0.043 (6)	0.023 (5)	0.015 (4)	−0.014 (4)	−0.012 (4)
C4	0.032 (4)	0.039 (5)	0.025 (5)	0.015 (4)	−0.015 (4)	−0.015 (4)
C5	0.026 (4)	0.017 (4)	0.013 (4)	0.001 (3)	−0.007 (3)	−0.002 (3)
C6	0.026 (4)	0.017 (4)	0.020 (5)	−0.001 (3)	−0.006 (4)	−0.007 (3)
C7	0.030 (4)	0.028 (4)	0.024 (5)	−0.015 (3)	0.000 (4)	−0.010 (4)
C8	0.034 (4)	0.024 (4)	0.028 (5)	−0.014 (3)	−0.007 (4)	−0.014 (4)
C9	0.022 (4)	0.017 (3)	0.021 (4)	0.004 (3)	−0.009 (3)	−0.006 (3)
O1	0.025 (3)	0.031 (3)	0.018 (3)	0.002 (2)	−0.014 (2)	−0.008 (2)
O2	0.029 (3)	0.028 (3)	0.015 (3)	0.007 (2)	−0.008 (3)	−0.013 (2)
O3	0.024 (3)	0.022 (3)	0.020 (3)	0.002 (2)	−0.004 (2)	−0.007 (2)
O4	0.032 (3)	0.026 (3)	0.019 (3)	−0.003 (2)	−0.005 (3)	−0.016 (3)
O5	0.030 (3)	0.020 (3)	0.023 (3)	0.004 (2)	−0.011 (3)	−0.003 (2)
O6	0.023 (3)	0.026 (3)	0.019 (3)	0.007 (2)	−0.004 (2)	0.003 (2)
O7	0.020 (3)	0.017 (3)	0.029 (3)	−0.001 (2)	−0.004 (3)	−0.001 (2)

### Geometric parameters (Å, °)

**Table:**

Yb—O1	2.790 (6)	C3—H3	0.9300
Yb—O1^i^	2.315 (6)	C4—H4	0.9300
Yb—O2	2.314 (6)	C5—O4	1.249 (10)
Yb—O3	2.264 (6)	C5—O3	1.264 (10)
Yb—O4^ii^	2.209 (6)	C5—C6	1.497 (12)
Yb—O5	2.310 (6)	C6—C7	1.387 (13)
Yb—O6^iii^	2.321 (6)	C6—C8	1.395 (11)
Yb—O7	2.293 (5)	C7—C8^v^	1.377 (12)
Yb—Yb^i^	4.2315 (10)	C7—H7	0.9300
C1—O2	1.274 (10)	C8—H8	0.9300
C1—O1	1.277 (10)	C9—O6	1.245 (10)
C1—C2	1.473 (12)	C9—O5	1.269 (9)
C2—C3	1.390 (13)	C9—C9^iii^	1.550 (15)
C2—C4	1.401 (12)	O7—H7B	0.85
C3—C4^iv^	1.390 (13)	O7—H7A	0.85
			
O4^ii^—Yb—O3	98.9 (2)	O1—Yb—Yb^i^	30.57 (12)
O4^ii^—Yb—O7	102.6 (2)	C9^iii^—Yb—Yb^i^	163.60 (15)
O3—Yb—O7	141.9 (2)	O2—C1—O1	119.6 (8)
O4^ii^—Yb—O5	79.5 (2)	O2—C1—C2	118.4 (8)
O3—Yb—O5	145.4 (2)	O1—C1—C2	121.8 (7)
O7—Yb—O5	70.19 (19)	C3—C2—C4	118.5 (8)
O4^ii^—Yb—O2	159.4 (3)	C3—C2—C1	120.3 (8)
O3—Yb—O2	85.5 (2)	C4—C2—C1	121.1 (8)
O7—Yb—O2	85.2 (2)	C4^iv^—C3—C2	120.5 (9)
O5—Yb—O2	85.5 (2)	C4^iv^—C3—H3	119.8
O4^ii^—Yb—O1^i^	82.5 (2)	C2—C3—H3	119.8
O3—Yb—O1^i^	80.7 (2)	C3^iv^—C4—C2	121.0 (8)
O7—Yb—O1^i^	71.5 (2)	C3^iv^—C4—H4	119.5
O5—Yb—O1^i^	132.4 (2)	C2—C4—H4	119.5
O2—Yb—O1^i^	118.1 (2)	O4—C5—O3	124.2 (7)
O4^ii^—Yb—O6^iii^	79.2 (2)	O4—C5—C6	117.8 (7)
O3—Yb—O6^iii^	75.4 (2)	O3—C5—C6	118.0 (7)
O7—Yb—O6^iii^	139.4 (2)	C7—C6—C8	118.8 (8)
O5—Yb—O6^iii^	70.34 (19)	C7—C6—C5	120.4 (8)
O2—Yb—O6^iii^	82.5 (2)	C8—C6—C5	120.7 (8)
O1^i^—Yb—O6^iii^	147.1 (2)	C8^v^—C7—C6	121.0 (7)
O4^ii^—Yb—O1	150.1 (2)	C8^v^—C7—H7	119.5
O3—Yb—O1	70.77 (19)	C6—C7—H7	119.5
O7—Yb—O1	74.9 (2)	C7^v^—C8—C6	120.2 (9)
O5—Yb—O1	125.1 (2)	C7^v^—C8—H8	119.9
O2—Yb—O1	50.14 (19)	C6—C8—H8	119.9
O1^i^—Yb—O1	68.4 (2)	O6—C9—O5	127.2 (8)
O6^iii^—Yb—O1	122.4 (2)	O6—C9—C9^iii^	117.0 (8)
O4^ii^—Yb—C9^iii^	72.4 (2)	O5—C9—C9^iii^	115.7 (8)
O3—Yb—C9^iii^	96.2 (2)	O5—C9—Yb^iii^	166.7 (6)
O7—Yb—C9^iii^	120.2 (2)	C9^iii^—C9—Yb^iii^	76.1 (5)
O5—Yb—C9^iii^	50.05 (19)	C1—O1—Yb^i^	165.8 (6)
O2—Yb—C9^iii^	87.2 (2)	C1—O1—Yb	82.4 (5)
O1^i^—Yb—C9^iii^	154.0 (2)	Yb^i^—O1—Yb	111.6 (2)
O6^iii^—Yb—C9^iii^	21.0 (2)	C1—O2—Yb	104.7 (5)
O1—Yb—C9^iii^	135.19 (19)	C5—O3—Yb	140.5 (5)
O4^ii^—Yb—Yb^i^	120.04 (17)	C5—O4—Yb^ii^	157.0 (6)
O3—Yb—Yb^i^	72.20 (14)	C9—O5—Yb	117.5 (5)
O7—Yb—Yb^i^	69.81 (14)	C9—O6—Yb^iii^	117.0 (5)
O5—Yb—Yb^i^	138.46 (14)	Yb—O7—H7B	123.8
O2—Yb—Yb^i^	80.49 (15)	Yb—O7—H7A	118.8
O1^i^—Yb—Yb^i^	37.81 (15)	H7B—O7—H7A	107.3
O6^iii^—Yb—Yb^i^	144.33 (15)		
			
O2—C1—C2—C3	−10.1 (13)	C2—C1—O2—Yb	−155.3 (6)
O1—C1—C2—C3	174.7 (9)	O4^ii^—Yb—O2—C1	161.8 (6)
O2—C1—C2—C4	165.3 (8)	O3—Yb—O2—C1	58.4 (5)
O1—C1—C2—C4	−9.8 (12)	O7—Yb—O2—C1	−84.5 (5)
C4—C2—C3—C4^iv^	1.1 (16)	O5—Yb—O2—C1	−155.0 (5)
C1—C2—C3—C4^iv^	176.7 (8)	O1^i^—Yb—O2—C1	−18.5 (6)
C3—C2—C4—C3^iv^	−1.1 (16)	O6^iii^—Yb—O2—C1	134.3 (5)
C1—C2—C4—C3^iv^	−176.7 (8)	O1—Yb—O2—C1	−10.2 (5)
O4—C5—C6—C7	−152.4 (9)	C9^iii^—Yb—O2—C1	154.9 (5)
O3—C5—C6—C7	27.6 (12)	Yb^i^—Yb—O2—C1	−14.2 (5)
O4—C5—C6—C8	30.4 (13)	O4—C5—O3—Yb	30.8 (14)
O3—C5—C6—C8	−149.6 (9)	C6—C5—O3—Yb	−149.2 (7)
C8—C6—C7—C8^v^	−1.5 (16)	O4^ii^—Yb—O3—C5	−46.7 (9)
C5—C6—C7—C8^v^	−178.8 (8)	O7—Yb—O3—C5	−170.7 (8)
C7—C6—C8—C7^v^	1.5 (16)	O5—Yb—O3—C5	37.6 (11)
C5—C6—C8—C7^v^	178.8 (8)	O2—Yb—O3—C5	113.0 (9)
O2—C1—O1—Yb^i^	171.7 (17)	O1^i^—Yb—O3—C5	−127.6 (9)
C2—C1—O1—Yb^i^	−13 (3)	O6^iii^—Yb—O3—C5	29.6 (9)
O2—C1—O1—Yb	−16.0 (7)	O1—Yb—O3—C5	162.2 (9)
C2—C1—O1—Yb	159.1 (7)	C9^iii^—Yb—O3—C5	26.3 (9)
O4^ii^—Yb—O1—C1	−164.4 (5)	Yb^i^—Yb—O3—C5	−165.5 (9)
O3—Yb—O1—C1	−90.6 (5)	O3—C5—O4—Yb^ii^	50.2 (19)
O7—Yb—O1—C1	106.3 (5)	C6—C5—O4—Yb^ii^	−129.7 (12)
O5—Yb—O1—C1	54.5 (5)	O6—C9—O5—Yb	−166.9 (7)
O2—Yb—O1—C1	9.9 (4)	C9^iii^—C9—O5—Yb	9.8 (12)
O1^i^—Yb—O1—C1	−178.0 (6)	Yb^iii^—C9—O5—Yb	160 (2)
O6^iii^—Yb—O1—C1	−33.1 (5)	O4^ii^—Yb—O5—C9	69.9 (6)
C9^iii^—Yb—O1—C1	−11.5 (6)	O3—Yb—O5—C9	−20.5 (8)
Yb^i^—Yb—O1—C1	−178.0 (6)	O7—Yb—O5—C9	177.6 (7)
O4^ii^—Yb—O1—Yb^i^	13.6 (5)	O2—Yb—O5—C9	−95.9 (6)
O3—Yb—O1—Yb^i^	87.4 (3)	O1^i^—Yb—O5—C9	139.5 (6)
O7—Yb—O1—Yb^i^	−75.7 (3)	O6^iii^—Yb—O5—C9	−12.3 (6)
O5—Yb—O1—Yb^i^	−127.5 (2)	O1—Yb—O5—C9	−128.6 (6)
O2—Yb—O1—Yb^i^	−172.1 (4)	C9^iii^—Yb—O5—C9	−5.8 (7)
O1^i^—Yb—O1—Yb^i^	0.000 (2)	Yb^i^—Yb—O5—C9	−166.1 (5)
O6^iii^—Yb—O1—Yb^i^	144.9 (2)	O5—C9—O6—Yb^iii^	−169.3 (7)
C9^iii^—Yb—O1—Yb^i^	166.5 (2)	C9^iii^—C9—O6—Yb^iii^	14.1 (12)
O1—C1—O2—Yb	19.9 (9)		

Symmetry codes: (i) −*x*+1, −*y*, −*z*+2; (ii) −*x*+1, −*y*+1, −*z*+2; (iii) −*x*, −*y*+1, −*z*+2; (iv) −*x*+1, −*y*, −*z*+1; (v) −*x*+2, −*y*+1, −*z*+1.

### Hydrogen-bond geometry (Å, °)

**Table:**

*D*—H···*A*	*D*—H	H···*A*	*D*···*A*	*D*—H···*A*
O7—H7A···O3^i^	0.85	1.92	2.751 (6)	167
O7—H7B···O2^vi^	0.85	1.91	2.754 (6)	178

Symmetry codes: (i) −*x*+1, −*y*, −*z*+2; (vi) −*x*, −*y*, −*z*+2.
